# Helical vasculogenesis driven by cell chirality

**DOI:** 10.1126/sciadv.adj3582

**Published:** 2024-02-21

**Authors:** Haokang Zhang, Tasnif Rahman, Shuhan Lu, Alejandro Pablo Adam, Leo Q. Wan

**Affiliations:** ^1^ Department of Biomedical Engineering, Rensselaer Polytechnic Institute, Troy, NY 12180, USA.; ^2^ Center for Biotechnology and Interdisciplinary Studies, Rensselaer Polytechnic Institute, Troy, NY 12180, USA.; ^3^ Department of Molecular and Cellular Physiology, Albany Medical College, Albany, NY 12208, USA.; ^4^ Department of Ophthalmology, Albany Medical College, Albany, NY 12208, USA.; ^5^ Department of Biological Sciences, Rensselaer Polytechnic Institute, Troy, NY 12180, USA.; ^6^ Center for Modeling, Simulation and Imaging in Medicine, Rensselaer Polytechnic Institute, Troy, NY 12180, USA.

## Abstract

The cellular helical structure is well known for its crucial role in development and disease. Nevertheless, the underlying mechanism governing this phenomenon remains largely unexplored, particularly in recapitulating it in well-controlled engineering systems. Leveraging advanced microfluidics, we present compelling evidence of the spontaneous emergence of helical endothelial tubes exhibiting robust right-handedness governed by inherent cell chirality. To strengthen our findings, we identify a consistent bias toward the same chirality in mouse vascular tissues. Manipulating endothelial cell chirality using small-molecule drugs produces a dose-dependent reversal of the handedness in engineered vessels, accompanied by non-monotonic changes in vascular permeability. Moreover, our three-dimensional cell vertex model provides biomechanical insights into the chiral morphogenesis process, highlighting the role of cellular torque and tissue fluidity in its regulation. Our study unravels an intriguing mechanism underlying vascular chiral morphogenesis, shedding light on the broader implications and distinctive perspectives of tubulogenesis within biological systems.

## Teaser

Endothelial cells spontaneously form a right-handed helix of biasedly aligned cells, driven by intrinsic cellular handedness.

## INTRODUCTION

The morphogenesis of tubular tissues or organs is a fundamental process occurring in early development across organisms (*1*–*3*). These structures, such as the embryonic heart tubes in vertebrates (*2*, *4*, *5*) and the embryonic hindgut in *Drosophila* (*3*, *5*, *6*), are among the first to exhibit left-right (LR) symmetry breaking. Previous studies have demonstrated that the LR asymmetry during tubular morphogenesis can originate from cellular chirality (*2*, *3*, *7*–*9*). This intrinsic LR asymmetric property of cells is implicated in various processes across multiple morphological scales (*2*, *3*, *10*–*15*), with the cellular chiral bias exhibiting substantial consistency with the handedness displayed at multicellular/tissue level (*2*, *10*, *13*, *16*). While impressive progress has been made in understanding the role of cell chirality in the tubular morphogenesis of epithelial or cardiac tissues, the comprehension of LR asymmetry in regular endothelial blood vessels, another typical tubular structure, remains very limited.

In the native environment, blood vessels are covered primarily with endothelial cells at the inner surface of the cylindrical wall, with their apical side facing toward the center of the vessel. These cells form a tight endothelial lumen separating the intravascular environment from the extravascular space and allowing the dynamic regulation of vascular permeability (*17*–*19*). Notably, we have previously reported that the endothelial cells have a strong clockwise (CW) chiral bias (*10*, *11*, *20*). The neutralization or randomization of this CW bias resulted in junctional disruptions and elevation of endothelial permeability due to the inability to form intact junctions between cells with heterogeneous chirality (*10*). Despite the demonstrated regulatory roles of cell chirality in endothelial functions, current research on the morphological and biophysical aspects of tubular vessels has primarily focused on cell behaviors or cues that are longitudinal (*21*–*23*), circumferential (*23*, *24*), or perpendicular (*25*) to the curved substrate surface, ignoring the nature of cell handedness or associated biases in cell alignments and mechanical forces.

The exhibition of LR asymmetry or chirality of a system typically requires two predetermined axes, such as the apical-basal (AB) axis and the front-rear (FR) axis (*12*, *14*). However, the polarization of the tissue along the longitudinal axis is not always clear, such as in a developing heart, where no directional cue is involved. Blood flow is directional, but it is absent for the initial phase of vascular development and angiogenesis and, therefore, cannot fully explain the cell polarization. This raises the question of whether and how the chiral property of endothelial cells can manifest morphologically on the tubular substrate with only the AB axis present. Although blood vessels do not undergo tissue/organ scale asymmetrical morphogenesis like the chiral C-looping of heart tubes, a helical alignment of endothelial cells on the tubular geometries can be seen in multiple studies featuring in vitro three-dimensional (3D) vascular platforms (*26*–*28*), but this phenomenon has never been studied in detail, and the handedness has not been quantitatively assessed.

In this work, to remove any possible systemic effects from animal models, we are to examine the chiral morphogenesis of human umbilical vascular endothelial cells (hUVECs) by engineering an in vitro 3D vascular microfluidics platform. We found a right-handed helical cell alignment, and a similar helical morphology with an identical directional bias was observed in mouse vasculature samples. We show that altering cell chirality through activating protein kinase C (PKC) reverses the helical handedness while increasing tissue fluidity through regulating cell contractility or cell-cell adhesion attenuates the chiral morphology. Our numerical simulation with the cell vertex model (CVM) reveals that cellular chiral torques drive the chiral morphogenesis we observed in in vitro and in vivo systems. In addition, we show that altering the flow profile and modulating substrate stiffness have no effect on the observed vascular handedness while reducing vascular surface curvature significantly disrupts the cell alignment bias. Our findings uncover a unique mechanism of LR asymmetry in tubular vascular morphogenesis, providing unique insights into the development and mechanics of the endothelial vascular system.

## RESULTS

### Endothelial cells exhibited chiral helical morphogenesis in in vitro vessels

**Fig. 1.**
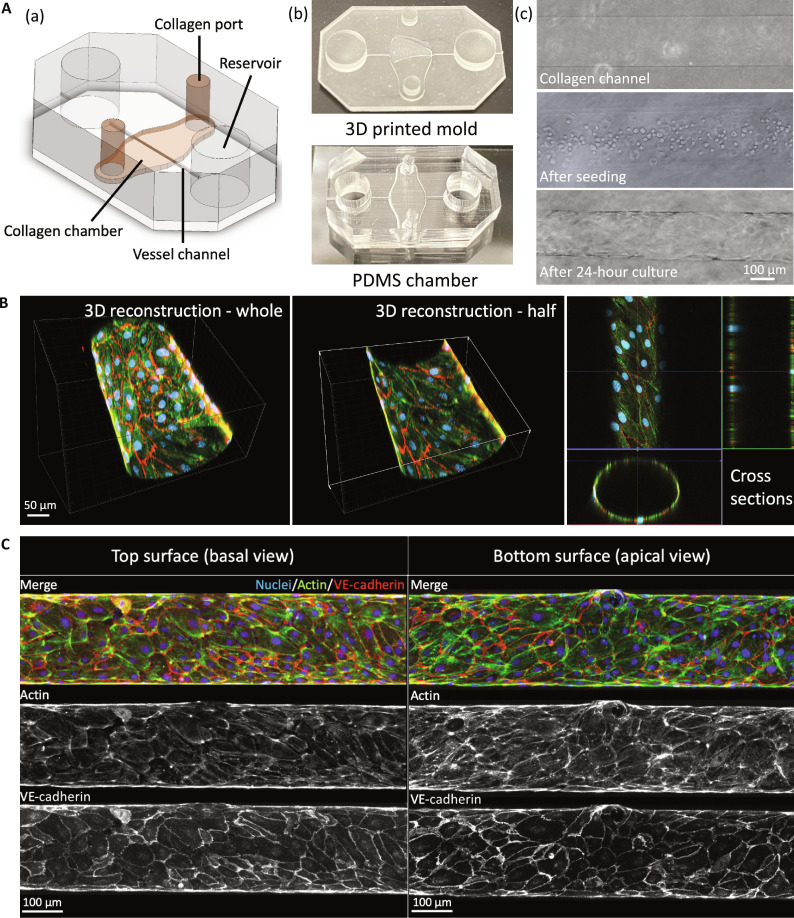
Confocal imaging of the in vitro vessels. (**A**) (a) Schematic of the polydimethylsiloxane (PDMS) microfluidic chamber. (b) Top: Three-dimensional (3D) printed master. Bottom: PDMS molded after the master and bonded to a glass coverslip. (c) Top: Molded collagen channel. Middle: Collagen channel after cell seeding. Bottom: Cell-seeded collagen channel after 24-hour culture. (**B**) 3D reconstruction of *z*-stack scanning of an in vitro vessel using a confocal microscope. The vessel was co-stained for actin (green), VE-cadherin (red), and nuclei (blue). Left: The 3D reconstruction of the whole vessel. Middle: The 3D reconstruction of the bottom half of the vessel. Right: Cross sections of the vessel. (**C**) 2D projections of the *z*-stack image sets separated into the top surface (basal view) and bottom surface (apical view) of the vessel. The images are presented as merged, actin channel, and VE-cadherin channel.

Using microfabrication techniques, we created polydimethylsiloxane (PDMS)–based in vitro vascular microfluidic devices, with hUVECs seeded into the needle-molded cylindrical channels (200 μm in diameter) in collagen extracellular matrix (ECM) (Fig. 1A) (*26*, *28*). After cells reached confluency on the collagen wall, an intact endothelial lumen was formed, resembling a typical vascular structure (Fig. 1B). The vascular constructs were cultured under a very gentle bidirectional oscillatory flow generated by a rocker to facilitate the lumen formation and nutrient exchange (*28*).

From *z*-stack confocal scanning of the immunofluorescence-stained vessels, we confirmed the formation of a confluent lumen structure inside the collagen gel, and the cell alignment exhibited an intriguing spiral or helical morphology with LR biases (Fig. 1B and movies S1 to S3). To better assess cell alignment on the lumen, we took the *z*-stack images and generated 2D projections of the top and bottom halves of the vessels separately (Fig. 1C). Please note that the 2D projections are presented as the view from a single observation direction. Therefore, the bottom projection is observed from the apical side of the lumen, while the top projection is observed from the basal side of the lumen. We observed a very clear cell alignment from top-left to bottom-right on the top surfaces while an opposite bottom-left to top-right cell alignment on the bottom surfaces. Given that the top and bottom projections represent views from opposite sides, our observations indicate a consistent asymmetric cell alignment direction across the entire lumen, and the vessel is a right-handed cellular helix along the longitudinal direction.

**Fig. 2.**
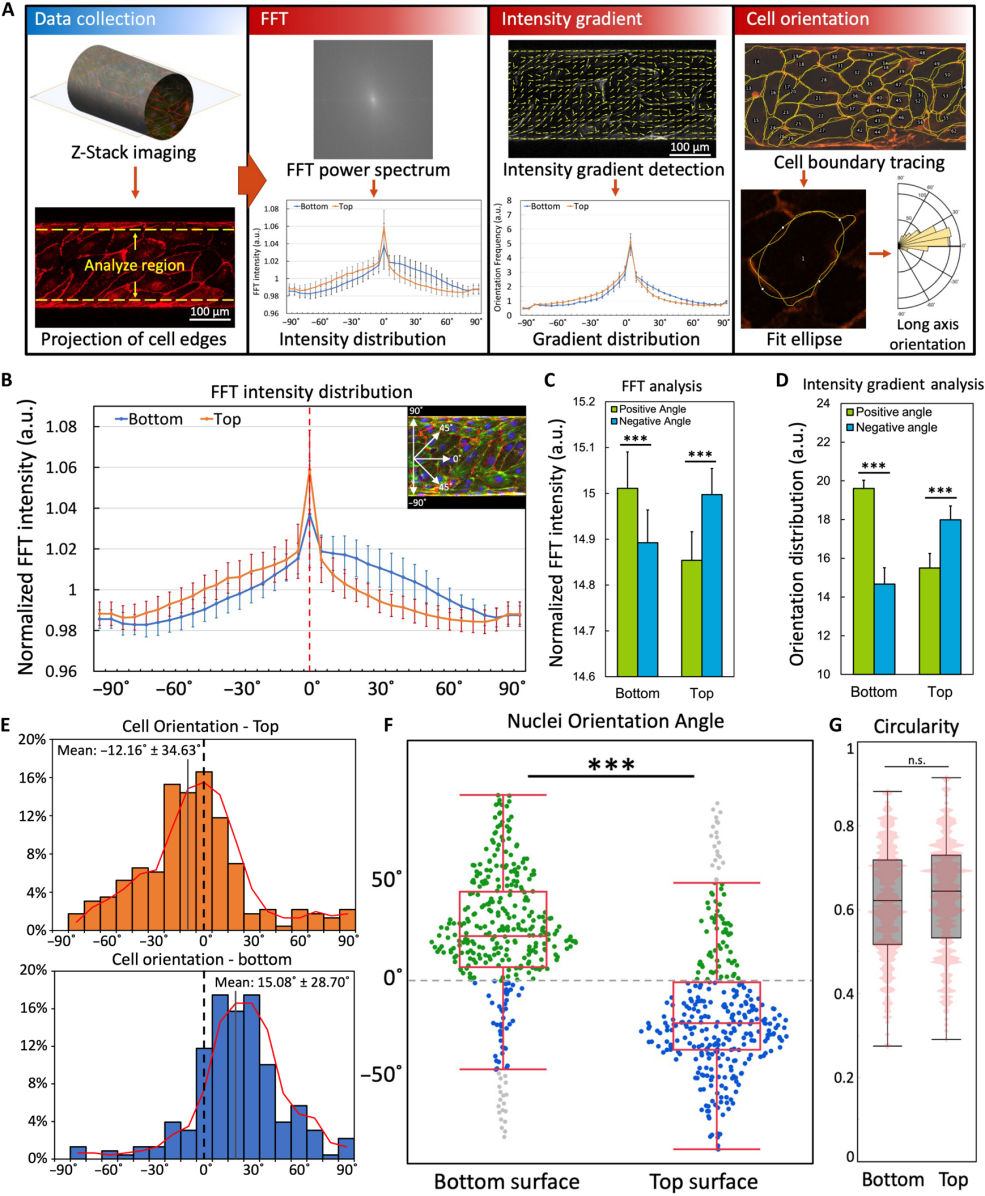
Characterization of the asymmetric helical cell morphology in the in vitro vessels. (**A**) Schematic of the data analysis workflow. The 2D projection of the VE-cadherin channel from *z*-stack imaging was processed using different methods. In the fast Fourier transform (FFT) method, the spatial images were converted into a 2D power spectrum, and then displayed as the angular distribution of spectrum intensity after radial summation. In the intensity gradient method, the cell alignment angle was determined by detecting the grayscale intensity gradient, and then the alignment angle frequencies were normalized and displayed as angular distribution. In the cell orientation assay, the cell boundaries were manually traced out and fitted by ellipses, and then the distribution of long-axis orientation angles was summarized. (**B**) Angular distribution of normalized FFT intensity of the projections and the FFT intensities were normalized to the mean value. *n* = 10 vessels, >5 image sets per vessel from different positions. Inset: The angular reference on the projection images. (**C**) Integrated FFT intensity distributed at positive angles (5° to 80°) versus negative angles (−80° to −5°), ***P < 0.001 by paired Student’s *t* test, *n* = 10 vessels, >5 image sets per vessel from different positions. (**D**) Normalized grayscale intensity gradient distributed at positive angles (5° to 80°) versus negative angles (−80° to −5°), ***P < 0.001 by paired Student’s *t* test, *n* > 10 vessels, >5 image sets per vessel from different positions. The raw values were normalized to the mean value. (**E**) Angular distribution of cell orientation on top and bottom surfaces (*n* = 10 vessels, >600 cells for each surface). The red line indicates the moving average. (**F**) Angular distribution of nuclei orientations at positive angles (green dot), negative angles (blue dot), and outliers (gray dot), ***P < 0.001 by Student’s *t* test, *n* = 10 vessels, >350 cells for each surface. (**G**) Circularity distribution of cells with pink contour representing distribution frequencies. n.s. represents no statistical difference, *n* = 10 vessels, >350 cells for each surface. a.u., arbitrary units.

To quantitatively assess the chiral morphogenesis in the in vitro microvessels, we characterized the asymmetric cell alignment in the projection images of VE-cadherin–labeled cell-cell boundaries with three different approaches (Fig. 2A). (i) Fast Fourier transform (FFT) analysis (*29*): The spatial images were converted into a 2D power spectrum in the frequency domain through FFT. Then, a radial summation of pixel intensity was applied to calculate the angular alignment distribution of cell boundaries. The amplitude in the 5° to 80° and −80° to −5° ranges were summed up and designated as the intensity for positive and negative angles, respectively. (ii) Intensity gradient analysis (*11*): The images were analyzed using a custom-written MATLAB program. First, the cell alignment angle was determined by detecting the grayscale intensity gradient, and then the alignment angle frequencies were categorized as positive or negative and compared with each other to determine the directional bias. (iii) Cell orientation analysis: The cell boundaries in the projection images were manually traced out and fitted by ellipses, and then the long-axis orientation angles were categorized as positive or negative, as described above. Please note that to reduce the interference from the edge deformation due to projection, only the central 150-μm wide region of the projection images was used for analysis (Fig. 2A).

From both FFT and intensity gradient analysis (Fig. 2, B to D), the vessels exhibited very stable and significant cell-cell boundary alignment bias toward the positive angles in the bottom projection images, while the top projection images showed a strong alignment bias toward the negative angles, demonstrating a consistent helical morphology across the lumen. As a more direct assessment method, the manual cell orientation analysis also outputted anticipated results (Fig. 2E), with the majority of cells on the bottom aligning toward the positive angles (15.08° ± 28.70°), while the cell on the top exhibited a preference for adopting a negative orientation (−12.16° ± 34.63°), and the overall helical pitch calculated from cell orientation was 2.3 ± 1.15 mm. Last, we quantified the nuclei orientation angles as further validation for the chiral bias. As demonstrated for the micropatterned cells (*30*), the asymmetric orientation of nuclei can also indicate the chiral bias of cells and show strong consistency with the cell alignment bias (*11*, *31*). The nuclei orientations on the engineered vessel also exhibited a significant bias toward the positive direction on the bottom and a corresponding bias toward the negative direction on the top (Fig. 2F), showing strong congruity with the other two methods. Moreover, the circularity of cells on the top and bottom projection images showed no statistical difference (Fig. 2G), indicating a consistent cellular morphology across the lumen. To simplify the explanation, we will only show the figures and the analyses of the lumen viewed from the apical side in further experiments.

In addition, to determine whether the right-handed helical cell alignment observed on a tubular substrate is common among endothelial cells, we extended the investigation to include endothelial cells from another tissue source, the human retina microvascular endothelial cells (hRMECs). We generated in vitro vessels using hRMECs and analyzed their handedness in vascular morphogenesis. Similar to vessels generated with hUVECs, the hRMEC vessels also exhibited a significantly biased cell alignment as a right-handed helix across the lumen (fig. S1A), with FFT analysis (fig. S1B) demonstrating a substantial bias in cell-cell boundary alignment toward positive angles, and cell orientation analysis revealing a dominant cell orientational bias toward positive angles (17.56° ± 41.67°; fig. S1, C and D). The overall helical pitch calculated from cell orientation was 2.0 ± 0.70 mm, which showed no statistical difference from the hUVEC vessels (fig. S1E). These findings indicate that hRMECs can also undergo spontaneous right-handed helical morphogenesis on a tubular substrate similar to hUVECs, which suggests that the asymmetrical tubular morphogenesis, characterized by the handed helical cell alignment, could be a common feature among various endothelial cell phenotypes.

Together, these results strongly demonstrated an asymmetric helical morphogenesis of endothelial cells in a cylindrical substrate resembling the vascular environment. The cells across the entire lumen aligned into a right-handed helix along the longitudinal direction of the in vitro vessels, which can only be explained by the intrinsic chiral property of the cell.

### Cellular components showed a consistent bias within the cell

The morphogenesis and functional regulation of the endothelial barrier heavily rely on the interdependent dynamics of the cadherin junction, actin structures, and actin-associated proteins, which have been demonstrated to couple with intracellular organizations (*32*, *33*). Therefore, we further examine whether the LR asymmetric cell alignment in the in vitro vessels is concomitant with the presence of the biased distribution of the cellular components mentioned above.

**Fig. 3.**
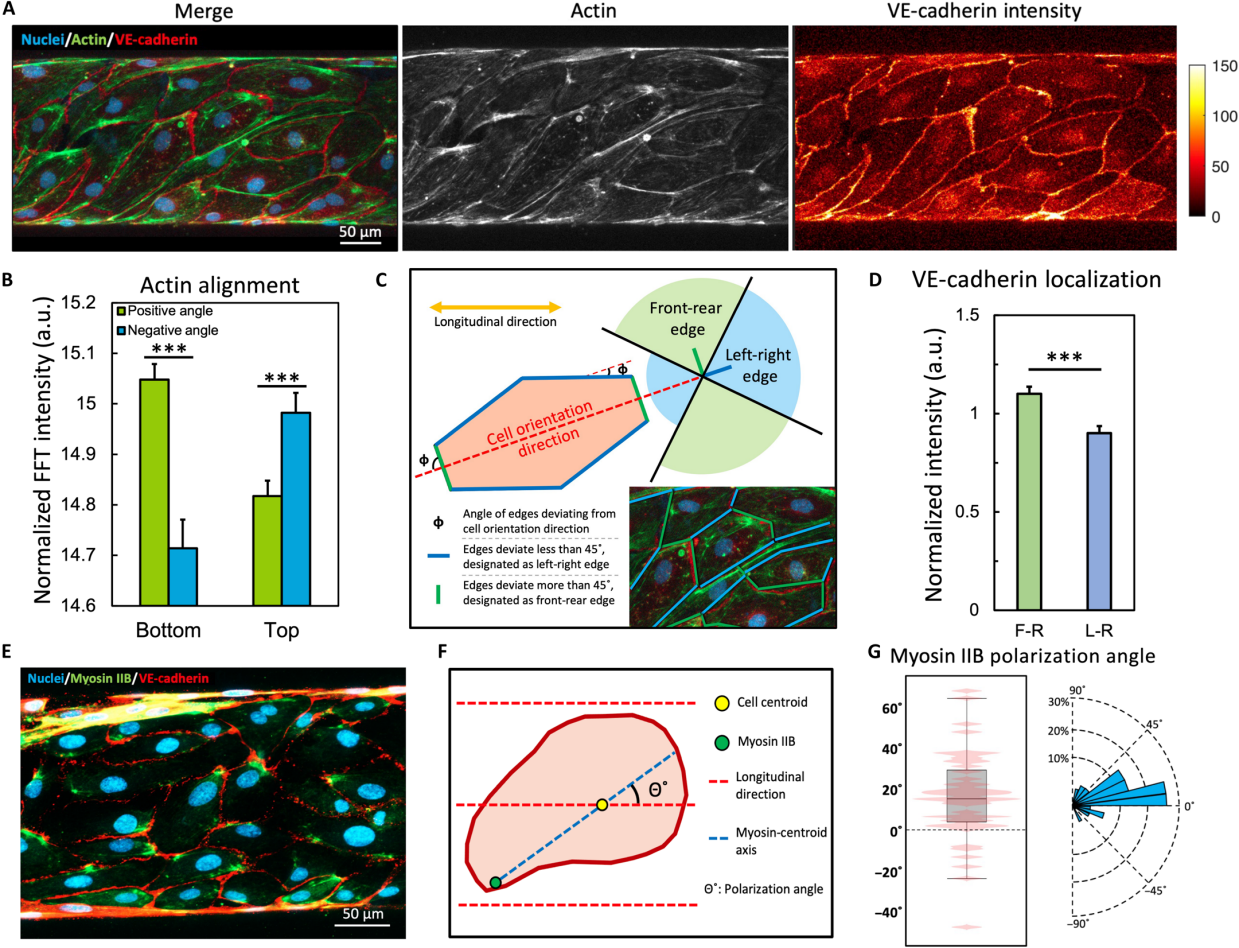
Asymmetric organization of intracellular structures. (**A**) Two-dimensional (2D) projection of the *z*-stack images showing co-staining of actin (green), nuclei (blue), and VE-cadherin (red). Images are shown as merged, actin channel, and VE-cadherin channel. The VE-cadherin channel is displayed as a fluorescence intensity heatmap processed by MATLAB. (**B**) Summation of normalized fast Fourier transform (FFT) intensity of actin aligning at positive angles (5° to 80°) versus negative angles (−80° to −5°), ***P < 0.001 by paired Student’s *t* test, *n* = 10 vessels, >5 image sets per vessel from different positions. (**C**) Schematic of categorizing cell edges into FR edges or LR edges. (**D**) Normalized fluorescence intensity of VE-cadherin localized to FR versus LR edges averaged by edge length, ***P < 0.001 by Student’s *t* test, *n* = 10 vessels, >5 image sets per vessel from different positions. (**E**) 2D projection of the *z*-stack images showing co-staining of myosin IIB (green), nuclei (blue), and VE-cadherin (red). (**F**) Schematic of calculating the myosin IIB polarization angle. (**G**) Left: Box plot showing the distribution of myosin IIB polarization angle, with pink contour representing frequencies. Right: Rose plot showing the radial distribution of myosin IIB polarization angle.

The actin filaments in the in vitro vessels exhibited a significant right-handed spiral morphology, with an alignment bias toward the positive direction when viewed from the apical surface, as characterized by the FFT analysis (Fig. 3, A and B), which is highly consistent with the cell alignment biases quantified in Fig. 2. The actin filaments spanned across the intercellular junctions and connected with the neighboring cells (Fig. 3A), eventually forming a uniform right-handed spiral alignment along the longitudinal direction of the vessel tube, which helps to mechanically stabilize and regulate endothelial junctions (*32*) and potentially contributes to cell elongation when cultured on a curved surface (*34*).

We next quantified the biased localization of VE-cadherin in the tubular endothelial sheet. VE-cadherin is the major component of the adherens junctions that coordinates junctional assembly following the contact of actin protrusions, mechanically stabilizes the cell-cell adhesion, and plays crucial roles in the maintenance of vascular integrity and permeability regulation (*17*, *35*). We generated the fluorescence intensity heatmap (Fig. 3A) and manually categorized the cell edges into LR edges or FR edges based on the orientational deviation angle from the individual cell orientation directions (Fig. 3C). Briefly, the edges aligned along the cell orientation direction (the blue region in Fig. 3C) were designated as LR edges (labeled in blue), while the edges aligning perpendicular to the cell orientation direction (the green region in Fig. 3C) were designated as FR edges (labeled in green). By quantifying the average fluorescence intensity along the length of the cell edges, a significantly higher intensity was found in the FR edges over LR edges (Fig. 3D). This result demonstrated a preferred localization of VE-cadherin on the FR edges of the cells that are perpendicular to the biased orientation/alignment direction of cells and actin in the in vitro vessels.

The nonmuscular myosin IIB is widely expressed in all vertebrates and participates in coordinating cell migration and adhesion, as well as establishing and stabilizing cell polarity (*36*, *37*). In endothelial cells, myosin IIB has been shown to localize to the rear end of the cell to facilitate tail retraction (*38*). From immunofluorescence staining, we confirm that the myosin IIB signal was localized to the end of elongated cells (Fig. 3E). To quantitatively assess the myosin IIB polarization, we measured the orientation angle of the polarization axis, defined as the line connecting the cell centroid and myosin IIB signal concentration (Fig. 3F). The results showed a biased orientation of the polarization axis toward the positive direction (16.84° ± 22.88°; Fig. 3G), demonstrating a consistent directional bias with the cell orientation. The polarization of myosin, together with that of VE-cadherin, to the FR end of the cell may contribute to the shrinkage of the FR edges, which, in turn, facilitates the proper elongation of the cells.

Together, these data indicated that the alignment of actin, the localization of VE-cadherin, and the polarization of myosin IIB work together to generate the corresponding asymmetrical cell alignment in the in vitro vessels (Fig. 2), further confirming the asymmetric helical morphogenesis of endothelial cells in the cylindrical substrate from a structural perspective.

### Mouse vasculature is helical with the same bias

**Fig. 4.**
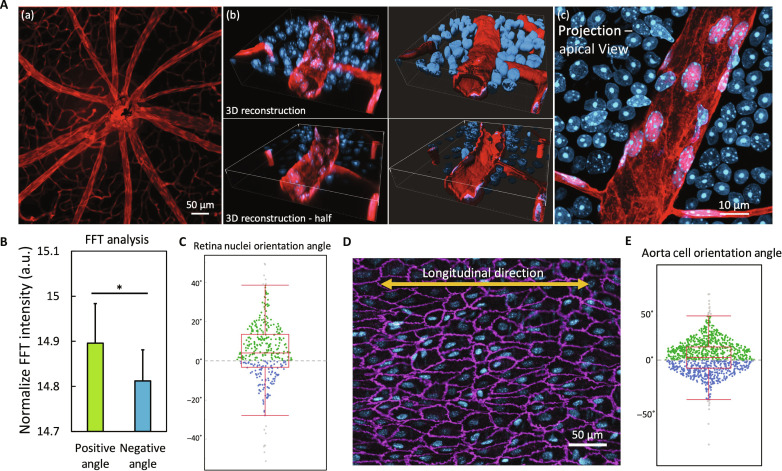
Imaging of mouse in vivo vasculatures. (**A**) (a) Low magnification image of the mouse retina samples, with endothelial cells labeled in tomato (red). (b) Three-dimensional (3D) reconstruction of the high magnification *z*-stack images of the mouse retina tissue. Left column: Maximum intensity projection. Right column: Normal shading view. (c) 2D projection of the *z*-stack images in (b). (**B**) Integrated fast Fourier transform (FFT) intensity distributed at positive angles (5° to 80°) versus negative angles (−80° to −5°), *P < 0.05 by paired Student’s *t* test, *n* = 5 retina samples, >6 image sets per vessel from different positions. (**C**) Angular distribution of nuclei orientations at positive angles (green dot), negative angles (blue dot), and outliers (gray dot), *n* = 5 retina samples, >200 cells in total. (**D**) In the high magnification image of a mouse aorta sample, the endothelial cell junction was stained with VE-cadherin (magenta) and cell nuclei with DAPI (blue). The longitudinal direction of the aorta is indicated by the orange arrow. (**E**) Angular distribution of nuclei orientations at positive angles (green dot) and negative angles (blue dot), *n* = 5 aorta samples, >1000 cells in total.

After characterizing and verifying the chiral morphogenesis in the in vitro vessels, we examined whether such asymmetric morphology naturally occurs in the in vivo vascular tissues. To this end, we imaged the microvessels ranging from 20 to 25 μm in diameter in mouse retina tissues, which is easily assessable for optical imaging without much physical manipulation. The endothelial cytoplasm was labeled with tdTomato, and nuclei with 4′,6-diamidino-2-phenylindole (DAPI) (Fig. 4A). Notably, the 2D projections viewed from the apical surface of the lumen revealed a significant cell alignment bias toward the positive direction, which is consistent with the morphology observed in the in vitro vessels (Fig. 1B). Quantification of cell alignment using the FFT method demonstrated a significantly higher normalized intensity at positive angles (Fig. 4B). Nuclei orientation angles also exhibited a strong bias toward the positive angles (4.30° ± 14.90°; Fig. 4C), indicating a right-handed helical alignment of cells and their nuclei. Moreover, to investigate cell alignment in larger vascular tissues, we conducted en face immunofluorescence staining of mouse aorta tissues and analyzed the cell orientation angles (Fig. 4, D and E). As expected, a biased cell orientation toward the positive direction was identified (3.02° ± 16.88°, Fig. 4E), indicating a similar right-handed helical cell alignment. Together, these findings confirmed that the asymmetric helical morphogenesis of the endothelium naturally exists in in vivo vessels, confirming the physiological relevance of our in vitro findings.

### PKC activation induced a dosage-dependent alteration of helical asymmetry in engineered vessels

**Fig. 5.**
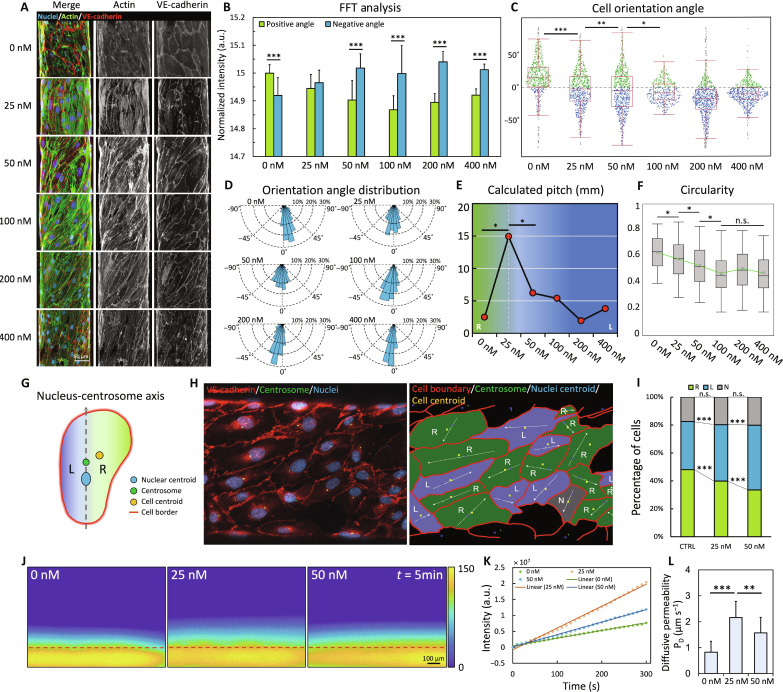
PKC activation altered the helical asymmetry in the in vitro vessels. (**A**) Projections of in vitro vessels. Vessels were co-stained for actin (green), nuclei (blue), and VE-cadherin (red). Images are presented as merged, actin channel, and VE-cadherin channel. (**B**) Summation of fast Fourier transform (FFT) intensity distributed at positive angles (5° to 80°) versus negative angles (−80° to −5°), ***P < 0.001 by paired Student’s *t* test, *n* > 6 vessels per group. (**C**) Angular distribution of cell long axis orientations at positive angles (green dot), negative angles (blue dot), outliers (gray dot), *n* > 6 vessels per group, >500 cells per group. *P < 0.05, **P < 0.01, and ***P < 0.001 by one-way ANOVA with Tukey’s HSD. (**D**) Rose plot showing the radial distribution of cell long axis orientations. (**E**) Calculated helical pitch of cell alignment. The green background represents the right-handed helix, and the blue background represents the left-handed helix, *P < 0.05 by one-way ANOVA with Tukey’s HSD (data presented as average, *n* > 6 vessels per group). (**F**) Variation of cell circularity. The mean value is indicated by a white triangle. *P < 0.05 by one-way ANOVA with Tukey’s HSD, *n* > 6 vessels per group, >500 cells per group. (**G**) Organelle positioning cell chirality characterization assay. (**H**) In situ characterization of cell chirality in the in vitro vessels. Left: Projection image of vessels co-stained for actin (green), nuclei (blue), and VE-cadherin (red). Right: Processed projection image labeled for cell boundary (red line), cell centrosome (green dot), nuclei centroid (blue dot), cell centroid (yellow dot), rightward biased cells (R, green), leftward biased cells (L, purple), non-biased cells (N, gray). (**I**) Percentages of rightward biased, leftward biased, and non-biased cells. ***P < 0.001, n.s. indicates no statistical difference by Student’s *t* test. (**J**) Intensity heatmaps of dextran diffusion. Vessel walls are indicated by red dashed lines. (**K**) FITC fluorescence influx into collagen with time. (**L**) Calculated diffusive permeability; **P < 0.01, ***P < 0.001 by Student’s *t* test, *n* > 6 vessels for each group.

We next asked the question of whether the asymmetric helical cell alignment in the vessels manifests as the result of cellular chirality. Using our previous finding that the activation of PKCα signaling through a small-molecule drug treatment reverses hUVEC chirality in a dosage-dependent manner (*10*), here, we first examined whether the helical handedness of the in vitro vessels can also be altered through PKC activation, by perfusing the vessels with medium supplemented with various concentrations of indolactam V (Indo V), a small-molecule PKC activator. At 25 nM Indo V, cell orientation in the in vitro vessels exhibited a significant alteration from a bias toward the positive direction (i.e., a right-hand helix, observed from the apical side of the lumen) in the untreated control to a non-biased morphology where the hUVECs aligned along the longitudinal direction of the vessel without significant deviation toward either side (Fig. 5, A to D), also evidenced by an increased helical pitch (Fig. 5E). At 50 nM Indo V, the cell orientation was shifted to the negative direction (i.e., a left-handed helix; Fig. 5, A to D) with a decreased pitch (Fig. 5E), demonstrating a complete reversal in the asymmetric helical cell alignment direction in the in vitro vessels. Notably, the reversal in the helical handedness was stably maintained with the treatment of higher concentrations (up to 400 nM) of Indo V (Fig. 5, A to E), and a decrease in cell circularity was observed with increasing Indo V concentration (Fig. 5F), similar to the previous reports in 2D (*10*).

To investigate the direct association of cellular chirality and the helical morphology in the in vitro vessels, we performed in situ cell chirality characterization by analyzing the relative positioning of the cell centroid and the nucleus-centrosome axis, as previously described (Fig. 5, G to I) (*10*, *15*, *20*, *39*). In the untreated control vessels (right-handed helix), we observed significantly more cells having a rightward centroid bias (48.0%, corresponds to CW chirality) relative to the nucleus-centrosome than leftward centroid bias [34.5%, corresponds to counterclockwise (CCW) chirality]. In vessels treated with 25 nM Indo V (non-biased longitudinal alignment), the cell centroid bias was lost, with 39.9% rightward-biased cells versus 40.4% leftward-biased cells. Last, in vessels treated with 50 nM Indo V (left-handed helix), the cell centroids exhibited a significant leftward bias (46.4%) over a rightward bias (33.5%), demonstrating a shift of the cellular chiral bias from CW to CCW. The percentage of non-biased cells in the three groups showed no statistical difference. Together, these results confirmed that the PKC activation mediated by Indo V treatment is sufficient to reverse the alignment bias of hUVECs in the in vitro vessels in a dosage-dependent manner, which is concomitant with the changes in dominant chirality, indicating that the right-handed helical morphogenesis in the 3D in vitro vessels originates from cellular chiral bias.

### PKC activation induced a non-monotonic variation of diffusive permeability in engineered vessels

We then explored the influence of PKC-regulated cell chirality on the function of the engineered vessels. Cell chirality has been demonstrated previously to be able to regulate endothelial permeability (*10*). Resulting from the poor junction formation between cells with unmatched chiral bias, the trans-endothelial permeability of 2D hUVEC monolayers exhibited a unique first increase then decrease trend of variation during PKC-mediated chirality reversal. To investigate whether such a non-monotonic pattern can also be observed during the PKC-mediated switching of the helix handedness of the in vitro vessels, we determined the diffusive permeability of the vessels under treatment with different concentrations of Indo V.

The vascular diffusive permeability was quantified by calculating the fluorescence influx rate into the collagen matrix upon perfusion with fluorescein isothiocyanate (FITC)–conjugated dextran solution (Fig. 5, J to L), following an established protocol in the field (*28*). In the range of 0 to 50 nM Indo V, the endothelial permeability first increased from 0.48 ± 0.1 μm s^−1^ (0 nM Indo V, right-handed helix) to 2.25 ± 0.8 μm s^−1^ (25 nM Indo V, non-biased cell alignment), then decreased to 1.6 ± 0.6 μm s^−1^ (50 nM Indo V, left-handed helix), demonstrating a non-monotonic variation of vascular permeability with increasing Indo V concentrations (Fig. 5, K and L). The observed pattern indicates that vascular permeability elevated to a peak when the cells lost the uniformed CW chirality and the vessels lost the right-handed helical morphology (at 25 nM Indo V; Fig. 5, A to D, I, and L) and then decreased as the cells fully reversed into a CCW chirality and the vessels adopted a left-handed helical morphology (at 50 nM Indo V; Fig. 5, A to D, I, and L). These findings further demonstrated that the asymmetric helical morphology of the in vitro vessels is associated with cellular chirality and that cell chirality regulates endothelial permeability (*10*) in a 3D vascular environment.

### Cell chirality determines the extent and direction of asymmetry in 3D vessels

**Fig. 6.**
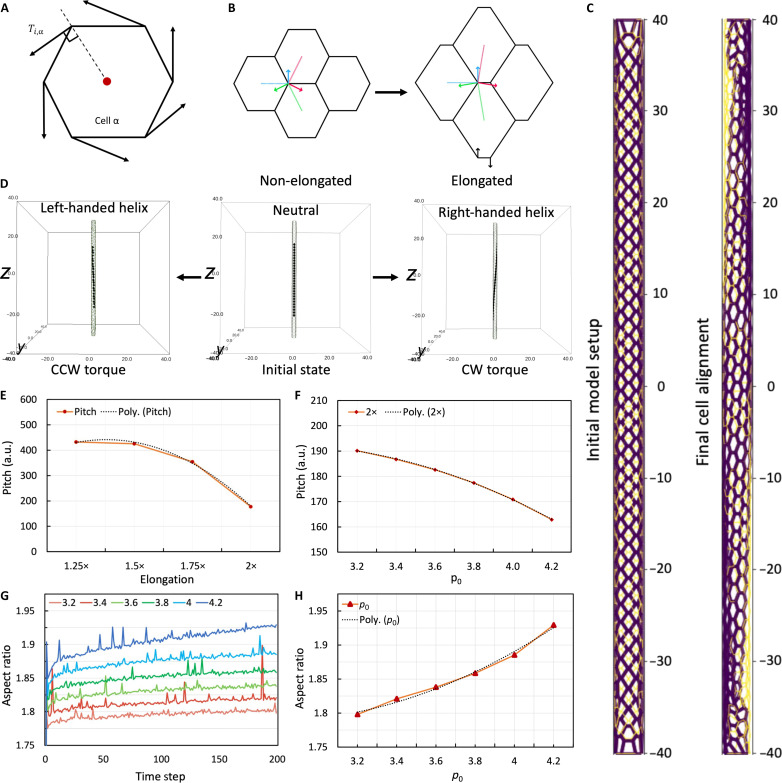
Computational simulation of cell alignment on a cylindrical tube. (**A**) Illustration of a hexagonal tissue showing the chiral forces acting on all associated vertices from the basal view, such that rotation direction is reversed. (**B**) Illustration of chiral torque forces on non-elongated cells (left) and elongated cells (right). The resultant force on the vertices is shown in the bottom vertices (black arrows). (**C**) Initial model microvessel tissue (left) and final model tissue (right). Purple cells with thick edges represent the top half of the initial cylindrical tissue, and thinner yellow edges represent cells on the bottom half of the initial cylindrical tissue. (**D**) Chiral alignment of microvessel upon application of counterclockwise (CCW; left) and clockwise (CW; right) torque on the initially symmetric tissue (middle). (**E**) Pitch calculation of cylindrical tissues under various elongations. (**F**) Pitch calculation of 2× elongated cylindrical tissues under various tissue fluidity (*p*_0_) values. (**G**) Average cell aspect ratio over time steps under different *p*_0_. (**H**) Average cell aspect ratio at endpoint under different *p*_0_.

To understand how cell chirality regulates the helical asymmetry in the in vitro vessels, we built a CVM for a cylindrical tube. Previous experiments suggest that individual cells are chiral (*40*, *41*) and that this rotational bias strongly correlates with multicellular asymmetry. Recently, cell chirality has been simulated in CVM to connect cellular chiral mechanical forces to the asymmetric cell alignment on micropatterns (*3*, *42*–*46*). Here, we extend the model to a 3D tubular shape and posit that the chiral torque is responsible for the observed asymmetric cell alignment on the cylindrical vessel. As endothelial cells that make up vessels tend to elongate along the vessel axis (*27*), we lengthened the simulated tissue by a factor of 1.25×, 1.5×, 1.75×, or 2× along the longitudinal axis. The elongation of the initially hexagonal cells leads to an imbalance in the chiral forces applied to each vertex, leading to the deformations needed for a biased cell alignment (Fig. 6, A to C). With a CW chiral torque, the cylindrical tissue takes on a right-handed helical shape, aligning the elongated cells in a similar manner to in vitro samples (Fig. 6C and movie S4). To mechanically validate our findings that the reversal of cell chirality (e.g., via PKC activation) can reverse the helix handedness of the in vitro vessels (Fig. 5), we simulated reversed the direction of the chiral torque and found that a CCW chiral torque resulted in a left-handed helical cell alignment (Fig. 6D). The level of tissue elongation affects the extent of asymmetry of the entire tissue. We found that more initial elongation leads to a more asymmetrically twisted vessel, characterized by a lower helical pitch (Fig. 6E). Moreover, during the course of the simulation, especially when a steady state is reached, no significant directional cell migration is observed, suggesting that chiral forces are sufficient to generate and maintain the handedness of the tubular tissue. Together, the computational simulation using CVM supports our in vitro findings that the right-handed helical cell alignment in the cylindrical vessels is driven by the chirality of endothelial cells.

### Tissue fluidity affects the chiral morphogenesis in the in vitro vessels

Using the computational model, we have previously shown that chiral morphogenesis can be regulated by cortical tension and cell-cell adhesion (*44*, *46*, *47*). Increasing cell contractility and reducing cell-cell adhesion forces lead to less fluid-like tissues and vice versa (*47*). Therefore, we were interested in determining whether such regulation exists within the vessels. Thus, in the simulations, we varied the cell shape index (*p*_0_), which is the ratio of the preferred cell perimeter and the square root of the preferred cell area, to quantify the effects of tissue fluidity on endothelial alignment. A larger *p*_0_ corresponds to stronger cell-cell adhesion and/or weaker cell cortical tension. Using our vascular CVM, we found that the more rigid tissues with lower *p*_0_ values have an increased helical pitch, which means that the cell alignment in these vessel tubes becomes less chiral compared to the more fluid-like tissues with higher *p*_0_ (Fig. 6F). We further computed the aspect ratio of cells and found that it is higher at all time points for tissues with a higher *p*_0_ (Fig. 6, G and H), indicating larger cellular deformation. These results are consistent with the previous computational observations in 2D (*44*, *46*).

**Fig. 7.**
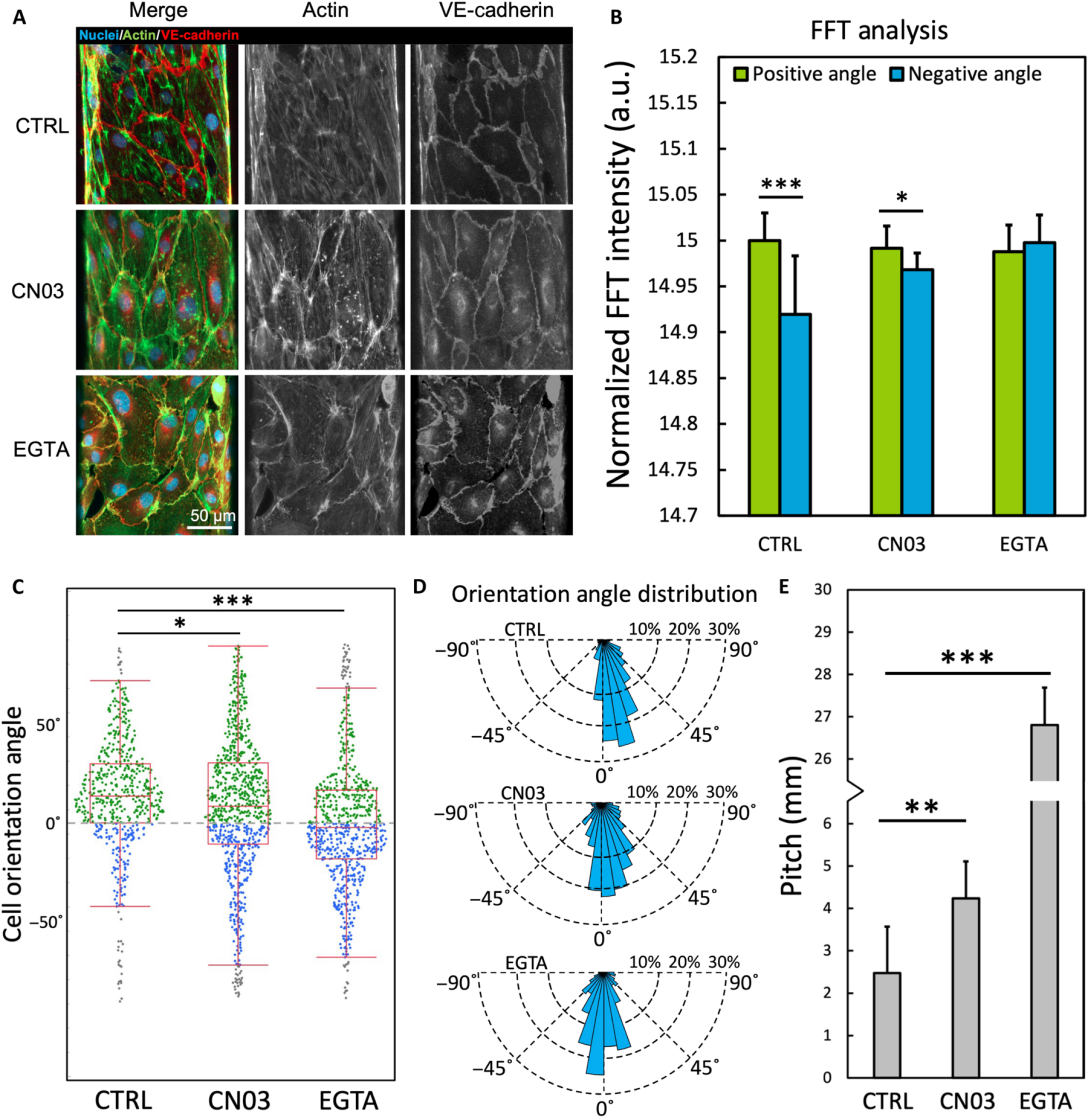
Tissue fluidity regulates the helical asymmetry in the in vitro vessels. (**A**) Two-dimensional projections of *z*-stack images of the in vitro vessels with CN03 or EGTA treatment. The vessels were co-stained for actin (green), nuclei (blue), and VE-cadherin (red). The images are presented as merged, actin channel, and VE-cadherin channel. (**B**) Summation of fast Fourier transform (FFT) intensity distributed at positive angles (5° to 80°) versus negative angles (−80° to −5°) with CN03 or EGTA treatment, *P < 0.01 and ***P < 0.001 by paired Student’s *t* test, *n* = 6 vessels per group, >5 image sets per vessel from different positions. (**C**) Angular distribution of cell long axis orientations with CN03 or EGTA treatment at positive angles (green dot), negative angles (blue dot), and outliers (gray dot), *n* = 6 vessels per group, >500 cells per group. *P < 0.05 and ***P < 0.001 by one-way ANOVA with Tukey’s HSD. (**D**) Rose plot showing the radial distribution of cell long axis orientations with CN03 or EGTA treatment. (**E**) The calculated helical pitch of cell alignment with CN03 or EGTA treatment (data presented as average by image sets, *n* = 6 vessels per group, >5 image sets per vessel from different positions, **P < 0.01 and ***P < 0.001 by one-way ANOVA with Tukey’s HSD).

To experimentally verify these findings from the computational simulations, we modulated the tissue fluidity of the in vitro vessels by promoting cell contractility with Rho signaling with the small-molecule Rho activator CN03 or limiting cell-cell adhesion with a calcium chelator, EGTA solution. Rho is a guanosine triphosphatase that plays important roles in actomyosin contractility and mechanotransduction (*48*), crucial for the regulation of stress fiber formation (*49*, *50*) and tissue fluidity homeostasis (*51*). Activating Rho will result in the elevation of cell contractility and, thus, the decrease in tissue fluidity. After supplementing CN03 into the vessel culture for 24 hours, we observed a significant attenuation of the asymmetric helical cell alignment, with weakened cell alignment and orientational bias (Fig. 7, A to D) and an increased helical pitch (Fig. 7E). Similarly, using EGTA treatment to destabilize intercellular junctions, reduce cell-cell adhesion, and thus decrease tissue fluidity, we found that the helical cell alignment and biased cell orientation were also significantly attenuated into a non-biased state (Fig. 7, A to D) with a much higher helical pitch (Fig. 7E). Consistent with its role destabilizing cell-cell junctions, we observed multiple gaps in the endothelial coverage induced by EGTA (Fig. 7A).

Together, these findings indicate that tissue fluidity can regulate the helical asymmetry of the in vitro vessels, with fluid-like tissues showing more significant chiral morphology, while more rigid tissue exhibited attenuated or non-biased morphology. Our results from both computational simulations and in vitro experiments demonstrated good coherence.

### The flow profile showed no effect of the helical asymmetry

Mature blood vessels in the native environment are subjected to unidirectional blood flow, and different flow profiles have been shown to have distinct effects on endothelial cell viability and alignment (*52*, *53*). To investigate whether flow profiles could affect the observed helical handedness in the in vitro vessels, we compared the morphology of vessels cultured under the oscillatory flow generated by a rocker and unidirectional flow generated by a syringe pump (see Materials and Methods).

The physiological wall shear stress (WSS) ranges from 10 to 20 dyn/cm^2^ in arteries and from 1 to 6 dyn/cm^2^ in veins (*54*). To ensure that the applied unidirectional flow induced physiological responses, the pumping rate was set to generate an approximate WSS of 10 dyn/cm^2^. Similar to vessels cultured under oscillatory flow, the 2D projections of vessels cultured under unidirectional flow showed a significant cell alignment bias and exhibited an overall right-handed helix along the vessel tube (fig. S2A). For vessels cultured under both conditions, FFT analysis (fig. S2B) revealed a substantial bias in cell-cell boundary alignment toward positive angles across the lumen, and cell orientation analysis also unveiled a dominant cell orientational bias toward positive angles (fig. S2, C and D). The overall helical pitch, calculated from cell orientation, showed no statistically significant difference between vessels cultured under different flow profiles (fig. S2E). These findings demonstrate that the flow profile did not affect the right-handed helical tubulogenesis of hUVECs in the in vitro vessels, indicating that the vascular helical asymmetry is a result of the intrinsic properties of the endothelial cells.

### Substrate stiffness showed no effect on vascular handedness

Substrate stiffness has a pivotal role in vascular morphogenesis and is closely associated with various vascular pathologies (*55*–*57*). We next assessed the impacts of modulating substrate stiffness on the vascular helical asymmetry. To adjust collagen stiffness without introducing variations in concentration-related biochemical properties (*58*, *59*), we supplemented genipin, a natural cross-linker, to the collagen solution at 0, 0.1, and 1 mM concentration, which is expected to yield an approximately 10-fold difference in Young’s modulus between consecutive groups (*58*).

For the in vitro vessels in all groups, the 2D projections of vessels showed a significant cell alignment bias, presented as a right-handed helix across the lumen with similar cell morphologies (fig. S3A). FFT analysis indicated a substantial bias in cell-cell boundary alignment toward positive angles for all groups (fig. S3B), and cell orientation analysis also showed a dominant cell orientational bias toward positive angles (14.24° ± 29.82° for 0 mM group, 15.84 ± 37.06° for 0.1 mM group, and 12.72° ± 34.73° for 1 mM group) with no statistical difference between any two groups (fig. S3, C and D). The calculated overall helical pitch for different stiffness groups showed no statistical difference either (2.48 ± 1.09 mm for the 0 mM group, 2.21 ± 0.83 mm for the 0.1 mM group, and 2.78 ± 0.91 mm for the 1 mM group; fig. S3E). These findings demonstrate that the substrate stiffness had no significant impact on the right-handed helical endothelial tubulogenesis in the in vitro vessels.

### Vascular curvature affects the helical handedness in in vitro vessels

The substrate surface curvature represents another critical mechanical factor with profound effects on guiding cell orientation and polarization (*34*, *60*). The alignment tendency of cells may contribute to the LR asymmetry in tubular vessels by guiding the establishment of the FR axis, eventually resulting in the observed helical handedness. Given these insights, we explored the influence of substrate curvatures on the helical handedness within the in vitro vessels, primarily by adjusting vessel diameter to modulate curvature magnitude. Through molding collagen with different-sized needles, we generated in vitro vessels with a diameter of 140, 200, and 250 μm.

For the in vitro vessels with diameters of 140 and 200 μm, the 2D projections of vessels showed significant right-handed cell alignment bias with similar cell morphologies (fig. S4A). However, for 250-μm vessels, the cell alignments exhibited notable inconsistency, with most cells orientating in different directions. FFT analysis revealed a substantial bias of cell boundary alignments toward positive angles in 140- and 200-μm vessels, while the significance was attenuated in 250-μm vessels (fig. S4B). From cell orientation analysis, a dominant cell orientational bias toward positive angles was found in 140-μm (15.41° ± 30.97°) and 200-μm (14.24 ± 29.82) vessels (fig. S4, C and D). However, in 250-μm vessels, the bias toward positive angles was notably weaker (1.18° ± 50.90°; fig. S4, C and D), presenting a disorganized non-biased morphology at the multicellular/tissue level. The overall helical pitch for different diameter groups also showed a similar trend (fig. S4E), with similar pitch values for 140-μm (2.28 ± 1.05 mm) and 200-μm (2.48 ± 1.10 mm) vessels and a significantly higher pitch value for 250-μm vessels (30.52 ± 0.51 mm). Together, these findings demonstrated that the reduction in substrate curvature disrupted the previously observed right-handed helical asymmetry in the in vitro vessels, resulting in cells orientating toward inconsistent directions.

## DISCUSSION

In this study, we revealed a spontaneous right-handed helical alignment of the endothelial cells in both in vitro microfluidics and in vivo tissues. Altering the cellular chiral bias with small-molecule drugs resulted in a dosage-dependent switch of the handedness of the engineered in vitro vessel, accompanied by a non-monotonic variation of vascular permeability. These results were then verified with numerical simulations using cell chirality-based CVM, with which we further showed that tissue fluidity could also affect the extent of vessel handedness. Then, by probing the potential effects of other relevant biomechanical factors, we found that flow profile and substrate stiffness had no effect on the vascular handedness while reducing surface curvature was able to disrupt the uniform cell alignment bias.

We believe that the right-handed cell alignment in the tubular vessels is driven by cellular chiral biases. By altering hUVEC chirality, we observed coherent results both in vitro (Fig. 5) and in silico (Fig. 6) that are compatible with the current understanding of endothelial chirality: (i) When cells have a CW chirality (0 μM Indo V, rightward organelle bias, CW chiral torque), the vessel tube exhibited a right-handed helical cell alignment and a low in vitro diffusive permeability; (ii) at an NC state (25 μM Indo V, neutral organelle bias), the vessel tube exhibited a non-biased longitudinal cell alignment with elevated in vitro permeability; (iii) when cells have a CCW chirality (≥50 μM Indo V, leftward organelle bias, CCW chiral torque), the vessel tube exhibited a left-handed helical cell alignment and a decreased in vitro permeability. These results, in different aspects, demonstrated that the helical morphology of the tubular vessels originated from cellular chirality and that such an intrinsic property could be mechanically and physiologically relevant to vascular architecture and functions. Other potential effects associated with PKC activation, such as changes in cell proliferation, are not likely to be involved, given the low-level activation conducted in this study (*10*). On the basis of our finding that the in vitro vessels cultured under bidirectional oscillatory flow and unidirectional laminar flow showed no difference in the helical handedness, and the fact that such biased cell alignment was also seen in previous in vitro studies under static conditions (*26*), we conclude that the flow profiles or even the presence of flow are not the determinant factors for the directionality of chiral vascular morphogenesis. As for the influence of other specific flow profiles, such as pulsatile flow, on the extent of chiral biases, we will leave it for future more detailed studies to evaluate the role of cell chirality in vascular disorders (*61*–*65*).

In this study, we have revealed a biophysical mechanism of tissue twisting regulated by cellular elongation. Endothelial elongation is widely implicated in the maintenance of vascular stability and functions (*66*). We found a more chiral cell alignment with a reduced helical pitch with increasing initial tissue elongation ratios (Fig. 6E). This is due to an increased resultant chiral force at the cell vertex that allows for a quicker and stronger emergence of the helical morphology of the vessel. In previous literature, endothelial cells have been shown to elongate longitudinally on tubular substrates, potentially resulting from the curvature sensing mechanisms that avoid bending of the actin cytoskeleton to minimize associated energy penalties (*27*, *34*, *60*, *67*). Therefore, it is possible that the preference for the minimal energy state at the longitudinal direction of the tube, together with the AB polarity, can further facilitate the formation of LR asymmetry of the tubular helix. By altering the vessel curvature, we have shown that cells on large vessels with small curvature exhibited an inconsistent orientational bias (fig. S4). A potential explanation is that the reduction in surface curvature weakened the substrate’s guiding effects on cell elongation along the longitudinal axis, which in turn resulted in the fluctuation in the LR axis, causing the cell with the same chirality to preferentially orientate in different directions. Further investigations, such as assessing the migrational profiles and actin dynamics of the cells during the vasculogenesis process, can help elucidate this mechanism.

We have demonstrated the mechanical significance of endothelium fluidity in chiral vascular morphogenesis. To our knowledge, this is the first study focused on the effects of tissue fluidity on vascular LR asymmetry. Our study showed that a more solid-like tissue resulted in a high helical pitch (less chiral) and reduced cell elongation (Figs. 6, F to H, and 7), similar to the previous findings from 2D simulations (*44*, *46*). More rigid tissues may inhibit cellular shear deformation or cell sliding (*42*) and therefore impair the process of chiral morphogenesis. Vasculature stiffening, characterized by the less elastic and more rigid vessel walls, is closely associated with the onset of vascular diseases such as atherosclerosis and pulmonary edema (*68*–*72*). In these cases, the decreased vessel fluidity may potentially affect endothelial chirality, which in turn may lead to subsequent disruption of vascular functions. Additional efforts will be necessary to determine the specific roles of cell chirality and tissue fluidity in vascular disease progressions.

In summary, the endothelial cells can spontaneously form a right-handed helical morphology in the in vitro tubular substrates and the in vivo vascular tissues. Reversal of the intrinsic cellular chirality altered the helical handedness of the vessels, which was supported by both in vitro experiments with PKC activation and computational simulations of switching chiral torque force directions. Our study revealed a unique mechanism of 3D vascular lumen morphogenesis driven by cell chirality, providing broad implications and unique insights into chirality in the development and diseases of the vascular system.

## MATERIALS AND METHODS

### Cell culture

The hUVECs (Lonza) and human retinal microvascular endothelial cells (hRMECs, Cell Systems) were used in this study. The hUVECs were cultured in the endothelial cell growth medium (EGM-Plus, Lonza) and used for experiments before passage 8, following the manufacturer’s instructions. hRMECs were cultured in the endothelial cell growth medium (classic complete medium, Cell Systems) and used for experiments before passage 12, following the manufacturer’s instructions. Both cell types were maintained at 37°C in a humidified incubator with 5% CO_2_, and the culture medium was refreshed every other day.

### Microfluidics

The microfluidic devices for culturing in vitro vessels were fabricated using soft lithography, following previous work by Polacheck *et al*. (*26*, *28*) with some modifications. Briefly, a 3D sketch of the mold was generated using SolidWorks (Dassault Systèmes), then 3D printed through a stereolithography 3D printer (Form 2, Formlabs) using clear printing resin (RS-F2-GPCL-04, Formlabs). PDMS (Sylgard 184, Dow-Corning) was mixed at a prepolymer to curing agent ratio of 10:1, and then cast onto the 3D printed molds and baked at 70°C for 6 hours. The cured PDMS was peeled off from the molds, trimmed, and punched out inlet/outlet and collagen ports with biopsy punches, and then surface-activated by plasma treatment for 90 s. Devices were then bonded to rectangular glass coverslips and subsequently treated with 0.01% poly-l-lysine (ScienCell) for 4 hours, 1% glutaraldehyde for 10 min, and water overnight on an orbital shaker. All devices were treated in a plasma-ozone cleaner (Novascan) for 15 min for sterilization, and then sterile steel acupuncture needles with different diameters (140, 200, and 250 μm, Tai-Chi Needles) coated with 1% bovine serum albumin (BSA) solution were inserted through the central chamber of devices. Rat tail collagen type I (Corning) solution was buffered with 10× phosphate-buffered saline (PBS), 1 N NaOH, and EGM-plus medium (Lonza), following instructions from the manufacturer, and brought to a final concentration of 4 mg/ml. The collagen solution was injected into the central chamber of devices and cross-linked under 37°C for 30 min. The growth medium was pipetted into the devices overnight, and needles were carefully removed to form 200-μm channels within the collagen gel. The collagen channels were rinsed with growth medium overnight on a rocker before cell seeding.

The hUVECs and hRMECs were trypsinized and resuspended into a density of 1 × 10^6^ cells/ml in their corresponding culture medium. A 75-μl cell suspension was introduced into the collagen channels, with 35 μl in one reservoir and 40 μl in the other, to establish a hydrostatic flow to drive the cells through the channel. The devices were immediately flipped upside down after adding cell suspension to allow cell attachment on the top side of the collagen channel for 5 min, followed by flipping the devices back and culture for another 5 min to facilitate cell attachment on the bottom. Then, the extra cells were washed off with a medium. Unless specified otherwise, all devices were generated with hUVECs and cultured under the gentle oscillatory flow on a rocker by rocking along the vessel axis for 24 to 48 hours until confluence. Medium is refreshed every day. All in vitro vessels mentioned in the results are generated with hUVECs unless specified otherwise.

### In vitro vessel culture with syringe pump-driven unidirectional flow

For experiment groups cultured under unidirectional flow, a hydrostatic pressure-driven flow generation module is modified from the protocol established by Polacheck *et al*. (*28*). Briefly, paraffin wax was melted in a boiling water bath and pipetted onto the device to cover the collagen ports. After the wax had cooled down and hardened, water-resistant nail polish was applied to seal the wax to the PDMS surface and it was allowed to dry in a biological safety cabinet. This step is to prevent large transmural pressure gradients with the flow. To set up the flow, the lid of a 100-mm petri dish was taken out and burnt a small hole using a soldering iron, then a tubing connector was glued onto the hole using an optical adhesive (Norland 81, Norland Products). Cell culture medium was filled into the tubing and syringe, with the tubing connected to the syringe via a male Luer lock connector and to a 25G needle via a female Luer lock connector. The syringe and tubing were mounted onto a syringe pump (Thermo Fisher Scientific). On the microfluidic device, a 300-μl micropipette tip with the tip cut off is glued onto one of the reservoirs using an optical adhesive (Norland 81, Norland Products) to create a pressure head. After cell seeding and static culture in the incubator for 4 hours, the device was moved to a deep-dish 100-mm petri dish. PBS was filled into the petri dish to just cover the top of the device, and an appropriate volume of medium was added to the pressure head to establish flow. The lid with the port was closed with a needle connected to the syringe pump inserted through the port and positioned inside the pressure head reservoir. The entire setup was placed in the incubator, and the syringe pump was started with the desired flow rate. The devices were cultured under unidirectional flow for 24 to 48 hours until confluence.

### Drug treatment

For PKC activation, 0 to 400 nM of Indo V (Sigma-Aldrich) was added to cell culture media 4 hours after cell seeding into the in vitro vessels. The control vessels were subjected to a similar amount of dimethyl sulfoxide (DMSO), which has no significant effects on cell chiral biases. For Rho activation, CN03 (5 μg/ml; Cytoskeleton Inc.) was added to cell culture media 4 hours after cell seeding into the in vitro vessels. The control group was subjected to a similar amount of DMSO, which had no significant effects. For EGTA treatment, 1 mM EGTA solution was added to cell culture media 4 hours after cell seeding into the in vitro vessels.

### Modulation of collagen hydrogel stiffness

To alter the stiffness of the ECM, a natural cross-linker, genipin, was supplemented into the collagen solution. Briefly, genipin powder (Thermo Fisher Scientific) was reconstituted in sterile distilled water to reach a final concentration of 10 mM as stock solution. To obtain a higher solubility, the vial containing the genipin solution was warmed to 37°C in a water bath and sonicated in an ultrasonic bath for 2 hours. The fully dissolved genipin stock solution was then sterile filtered using a 0.22-μm syringe filter and stored at −80°C before use. When cross-linking collagen hydrogel, the appropriate volume of genipin stock solution was mixed into the collagen solution to reach a final concentration of 0.1 or 1 mM, followed by injecting the collagen solution into the central chamber of devices containing the acupuncture needle and cross-linked under 37°C for 30 min. After needle removal, the collagen channel was sufficiently rinsed with endothelial growth medium on a rocker by rocking along the channel axis for 24 hours. All genipin stock solution used for the experiment was made within the week of collagen fabrication and went through no more than one freeze-thaw cycle to ensure potency. All devices used in the experiments are not supplemented with genipin unless specified otherwise.

### Immunofluorescence of in vitro vessels

All steps for the vessels below were conducted while the PDMS construct was placed on a rocker and rocking along the vessel axis. The vessels were fixed with 1% paraformaldehyde (PFA) in PBS++ (PBS with 1 mM CaCl_2_ and 0.5 mM MgCl_2_) under 37°C for 2 min and then with 4% PFA in PBS under 37°C for 8 min, followed by three times of PBS washes for 5 min each. The fixed vessels were permeabilized with 0.5% Triton X-100/PBS solution for 10 min, blocked with 10% normal goat serum in 0.1% Triton X-100/PBS solution for 1 hour, and then incubated with mouse anti–VE-cadherin antibody (Santa Cruz Biotechnology) and rabbit anti myosin IIB antibody (Cell Signaling Technology) or rabbit anti-pericentrin antibody (Abcam) overnight, followed by 1-hour incubation of Alexa Fluor 568 goat anti-mouse immunoglobulin G (IgG) H + L (Thermo Fisher Scientific) or Alexa Fluor 488 goat anti-rabbit IgG H + L (Thermo Fisher Scientific) for secondary labeling at room temperature. The vessels were also stained with Alexa Fluor 488 phalloidin (Invitrogen) for actin filaments and DAPI (Invitrogen) for nuclei under room temperature for 1 hour when needed. The samples were *z*-stack–imaged using a confocal microscope (LSM800, Carl Zeiss) with an air objective lens (20×, Carl Zeiss). The processing and presentation of the *z*-stack image sets were done using FIJI/ImageJ (NIH), Zen (blue edition, Carl Zeiss), and Imaris Viewer (Oxford Instruments).

### Organelle positioning analysis for in situ cell chirality characterization

The organelle positioning method for characterizing cell chirality has been described in our previous studies (*10*, *15*, *20*, *39*). As the axis orientated from the nucleus to the centrosome marks the FR axis of the cell, the two sides of this axis mark the left or right of the plane, and the position where the cell centroid falls indicates the chiral bias of the cell. Briefly, for each fluorescence image of the in vitro vessels labeled for VE-cadherin, nucleus, and pericentrin, manual segmentation was first performed to separate individual cells by following cell junction and nuclear contour in Photoshop CC (Adobe), and the positions of cell centroids and nuclei centroids were then determined using ImageJ (NIH). Last, the LR bias of each cell was determined on the basis of the positional bias of the cell centroid relative to the nucleus-centrosome axis. A cell with its centroid overlapping with the axis was considered non-biased.

### Immunofluorescence of mouse vessels

Mice with an endothelial-specific, tamoxifen-inducible tomato marker were maintained in accordance with protocols approved by the Institutional Animal Care and Use Committee at Albany Medical College. Cdh5-CreERT2 (PMID 20445537) and R26tdTomato (PMID 20023653) mice have been described previously (PMID 34138760). All mice received tamoxifen [2 mg of tamoxifen in 100 μl via intraperitoneal (ip) injection] at 6 to 9 weeks old for 5 consecutive days for Cre induction.

For retina extraction, the eyes were removed and placed in 4% PFA at 4°C after euthanasia, then transferred to PBS, and stored at 4°C until retina removal. After the removal of the cornea and lens, the edge of the retina was gently detached from the sclera, and four small cuts were made to the retina to allow flattening. The retinas were permeabilized with 1% BSA, 5% FBS, and 0.5% Triton X-100 in PBS solution for 1 hour at 37°C with gentle shaking using an orbital shaker. The samples were then incubated with DAPI (1 μg/ml; Invitrogen) in block solution for 1 hour at 37°C, followed by three PBS washes for 20 min each under room temperature, and mounted onto glass coverslips with Fluoromount-G (Southern Biotech). The samples were *z*-stack–imaged using a confocal microscope (LSM800, Carl Zeiss) with an oil object lens (63×/1.4, Carl Zeiss). The processing and presentation of the *z*-stack image sets were done using FIJI/Image J (NIH), Zen (blue edition, Carl Zeiss), and Imaris Viewer (Oxford Instruments).

The mouse aorta after extraction was fixed with 2% PFA at 4°C for 1 hour and stored in PBS at 4°C until use. For en face immunostaining of the aorta, the fixed aorta was rinsed in tris-buffered saline (TBS) and blocked overnight while rocking at 4°C in 10% goat serum in TBS with 0.3% Triton-X100 (TBSTX.3) with 300 mM glycine, then transferred to TBST.3, and washed for 30 min. The samples were incubated while rocking overnight at 4°C with rat anti-mouse CD144 antibody (BD Pharmingen) in 10% goat serum in TBSTX.3, followed by 2-hour washes in TBSTX.3 four times. For secondary staining, the aorta was incubated while rocking overnight at 4°C with Alexa Fluor Plus 647 donkey anti-rat antibody (Invitrogen) and DAPI (Invitrogen) in 10% goat serum in TBSTX.3, and washed in TBSTX.3 for 30 min, followed by 1-hour washes in TBSTX.3 three times and 10-min washes in TBS three times, all protected from light. For sample mounting, the aorta was carefully cut through the top arch and down the dorsal wall and the midline between intercostals using microscissors and ultrafine tweezers. The samples were placed face up (en face) on a microscope slide and completely opened up using ultrafine tweezers. Drops of mounting reagents (Southern Biotech) were placed on the sample as necessary, and then a coverslip with a 10-g weight was applied on top to flatten the aorta better. The samples were imaged using a confocal microscope (LSM800, Carl Zeiss) with an oil object lens (63×/1.4, Carl Zeiss).

### Permeability assay

The permeability of the in vitro vessels was measured following a previously reported method (*28*). Briefly, after the in vitro vessels reached confluency about 24 hours after seeding, the media was aspirated out from both reservoirs (fig. S1), and 50 μl of FITC-conjugated 40-kDa dextran solution was added to one of the reservoirs. Time-lapse imaging of the in vitro vessels with dextran flowing through was conducted using an inverted microscope (BZ-X700, Keyence) with a live cell culture chamber, and fluorescence images were acquired every 10 s for 5 min. The mass flux of dextran at the midplane of the vessels was then calculated on the basis of the intensity variation of the collagen region and vessel region with time in MATLAB (MathWorks). The diffusive permeability is calculated as *P*_d_ = (2*R*/*I*_0_)*I(*t*), where *P*_d_ is diffusive permeability, *R* is the radius of the in vitro vessel, *I*_0_ is the intensity of the vessel region, and *I*(*t*) is the intensity variation rate in the collagen matrix.

### Computational model description

To model the dynamics of endothelial cells within a microvessel, we used a CVM (*73*). In this model, cells are represented by 2D polygons in a gapless network resembling a net. The shared edges intersect at polygon vertices. The vertex positions and their associations with each other, forming the polygon edges, are used to define the cellular network and tissue shape. The vertex positions are subsequently updated during simulations to model the dynamics of the tissue. To model the microvessel, a vertex model-based tissue is generated as a finite cylinder—represented by a flat 2D polygon network wrapped around a 3D cylindrical shape. All model implementation was performed using the Python Tyssue Simulation Library (*74*).

#### Vertex model dynamics

Standard vertex model dynamics are used to simulate the time-dependent behavior (*47*, *73*). Briefly, two conservative forces are used to determine the total potential energy of the tissue: area elasticity and perimeter elasticity. Thus, the total energy of the system is defined as

$$E=\frac{K_{A}}{2}\sum_{\alpha =1}^{N}{\left(A_{\alpha }-A_{0}\right)}^{2}+\frac{K_{P}}{2}\sum_{\alpha =1}^{N}{\left(P_{\alpha }-P_{0}\right)}^{2}$$ (1)

Here, *K_A_* and *K_P_* are the elastic moduli associated with cell area and cell perimeter, respectively, *N* is the total number of cells, *A*_α_ and *P*_α_ are the area and perimeter of cell α, respectively, and *A*_0_ and *P*_0_ are the preferred area and perimeter for all cells, respectively.

Cell chirality is modeled as an active, rotational, torque-generating force applied to all polygon vertices as described previously (*44*, *46*). The forces applied are tangential to the cell centroid. Thus, the total force applied to any given vertex is the sum of all chiral forces acting on a vertex by the three cells associated with that vertex. This force on a given vertex *i* is given as

$${\vec{T}}_{i}=\sum_{\alpha }\nu_{\alpha }\left[\vec{n}\times \left({\vec{r}}_{i}-{\vec{r}}_{\alpha }\right)\right]$$ (2)

Here, the sum is taken over all cells α associated with the vertex *i*, ${\vec{r}}_{i}$ is the position vector of vertex *i*, ${\vec{r}}_{\alpha }$ is the position vector of cell α centroid, and $\vec{n}$ is the cell normal vector. Last, ν_α_ is the torque coefficient, which determines the strength and directionality of the chiral force, with negative values applying a CW force and positive values a CCW force. Adding the active chiral force to the area and perimeter elasticities gives the following time evolution of vertex positions (*44*, *75*)

$$\eta \frac{d{\vec{r}}_{i}}{\mathrm{dt}}=\frac{-\delta E\left(\{{\vec{r}}_{i}\}\right)}{\delta {\vec{r}}_{i}}+{\vec{T}}_{i}$$ (3)

Here, η represents the coefficient of viscous friction. A forward Euler method is used to solve this system of equations and simulate the dynamics of the tissue.

#### Initial vertex model tissue generation

The initial tissue is generated by distributing polygon centers on a cylindrical shape in 3D space, such that a Voronoi tessellation of the points leads to mostly hexagonal cells with an average area of ~1, based on arbitrary length units. The tissue is then stretched along the cylinder axis by applying a displacement to the vertices on either end of the tissue. CVM microvessels were elongated by factors of 1.25×, 1.5×, 1.75×, and 2×. We then minimize the energy of the tissue, which increases due to the applied strain, so that the areas of the cells are mostly homogenous and represent elongated hexagons. This is done to mimic the elongation of endothelial cells observed within a microvessel. No chiral forces are applied during this process.

#### Boundary conditions

Since our model tissue is a finite cylinder, the two ends of the cylinder represent circular boundaries on which the boundary vertices lie. Thus, appropriate boundary conditions are required to mimic in vitro microvessels. We assume that cells and their associated vertices on the boundary experience area and perimeter elasticity forces but do not experience chiral forces due to the irregular shapes of our in vitro microvessels. In addition, in in vivo vessels, chirality is not likely to propagate from the two ends to the middle region due to the presence of biological noises. We fix the boundary vertices in the *z* direction to avoid changes in the length of the cylindrical microvessel, as observed in vitro. Similarly, all vertices are fixed on the cylinder surface to maintain the integrity of the tissue surface.

#### Pitch calculation

The spiral pitch for CVM microvessels was calculated like what was done for in vitro microvessels. However, instead of using alignment of cell shape, we connected the cell centers of initially vertically aligned cells on the top surface of the tissue with their immediate vertical neighbors (cells labeled in black; Fig. 6D) and measured the angles that these connected lines made relative to the vertical *z* axis. The average angle was then taken for all lines connecting vertical neighbors. The average angle was then used to calculate the pitch such that

$$\text{tan}\left(\Theta \right)=\frac{c}{\text{pitch}}$$

where Θ is the average angle relative to the longitudinal axis, and *c* is the circumference of the microvessel cross section.

### Statistics

For all data generated using in vitro vessels, *n* describes the total number of technical replicates from ≥3 biological replicates of the corresponding experiment. For data generated using in vivo tissues, *n* describes the number of biological replicates. JMP 16 (SAS) and Excel (Microsoft) were used for statistical analysis. Data were presented as average ± SD unless indicated otherwise. One-way analysis of variance (ANOVA) with Tukey’s honestly significant difference (HSD) method for multiple comparisons was performed for dosage-dependent responses or multiple independent groups. Student’s *t* test (two-tailed) was performed for comparison between the two groups. Significant differences were performed at a confidence level of 0.05 for all statistical tests.
