# Supplementary material for: Helical vasculogenesis driven by cell chirality
Source: Sci Adv. 2024 Feb 21;10(8):eadj3582. doi: 10.1126/sciadv.adj3582 (PMC10881055; doi:10.1126/sciadv.adj3582)
Supplement: Supplementary file 1 — Figs. S1 to S4 Legends for movies S1 to S4 [file sciadv.adj3582_sm.pdf]

Science Advances
AAAS

Supplementary Materials for

Helical vasculogenesis driven by cell chirality

Haokang Zhang *et al.*

Corresponding author: Leo Q. Wan, wanq@rpi.edu

Sci. Adv. **10**, eadj3582 (2024)
DOI: 10.1126/sciadv.adj3582

The PDF file includes:

Figs. S1 to S4
Legends for movies S1 to S4

Other Supplementary Material for this manuscript includes the following:

Movies S1 to S4

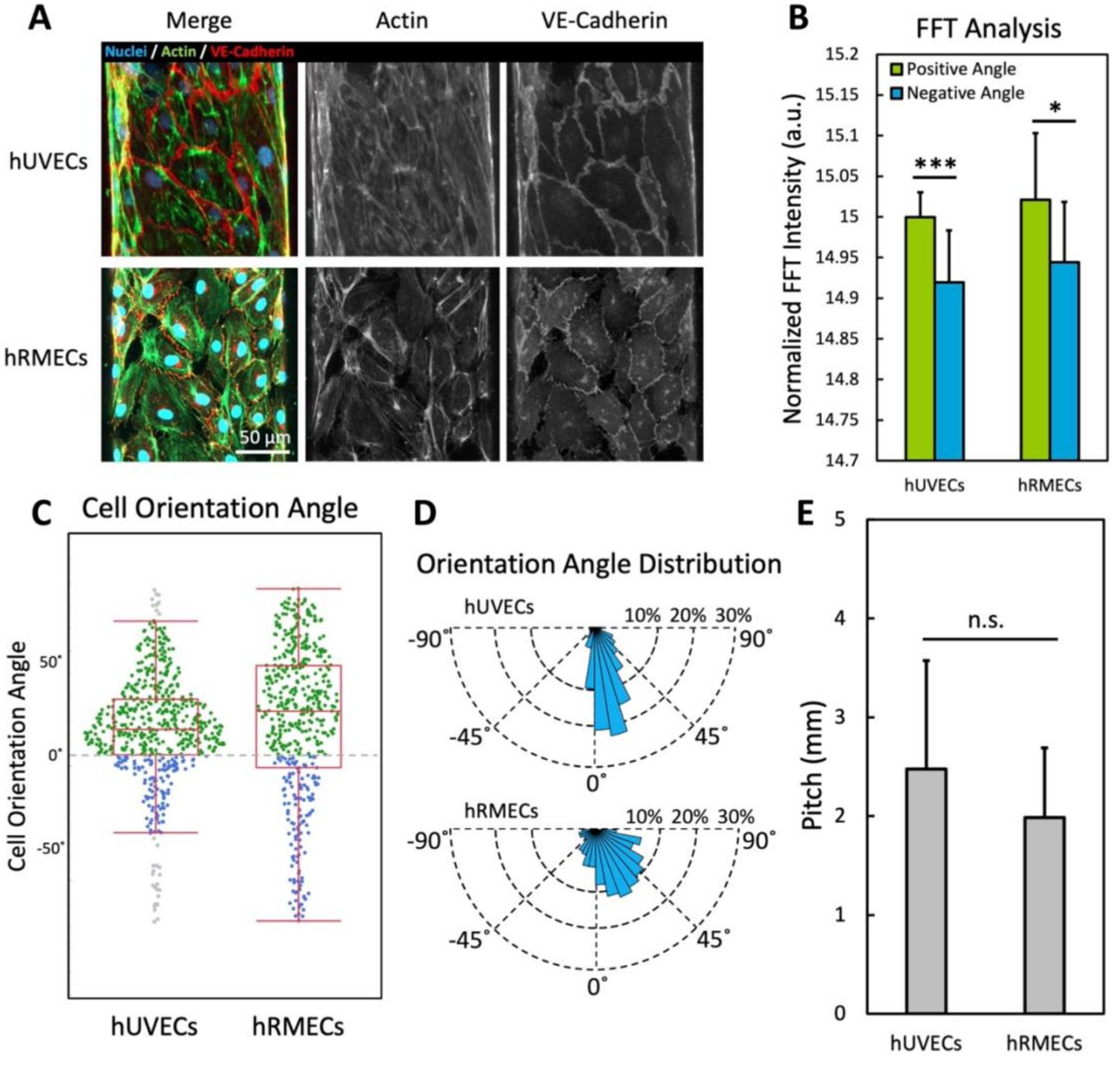

Figure S1. hRMECs exhibit right-handed helical asymmetry in the *in vitro* vessels. (A) 2D projections of z-stack images of the *in vitro* vessels generated with hUVECs or hRMECs. The vessels were co-stained for actin (green), nuclei (blue), and VE-Cadherin (red). The images are presented as merged, actin channel, and VE-Cadherin channel. (B) Summation of FFT intensity distributed at positive angles (5˚- 80˚) *vs.* negative angles (-80˚- -5˚) for vessels with hUVECs or hRMECs, $p^* < 0.05$ and $p^{***} < 0.001$ by paired student's t-test, n=5 vessels per group, >5 image sets per vessel from different positions. (C) Angular distribution of cell long axis orientations for hUVEC or hRMEC vessels at positive angles (green dot), negative angles (blue dot), and outliers (grey dot), n=5 vessels per group, >400 cells per group. (D) Rose plot showing the radial distribution of cell long axis orientations of hUVEC or hRMEC vessels. (E) The calculated helical

pitch of cell alignment for hUVEC or hRMEC vessels (data presented as average by image sets, n=5 vessels per group, >5 image sets per vessel from different positions). n.s. represents no statistical difference by student's t-test.

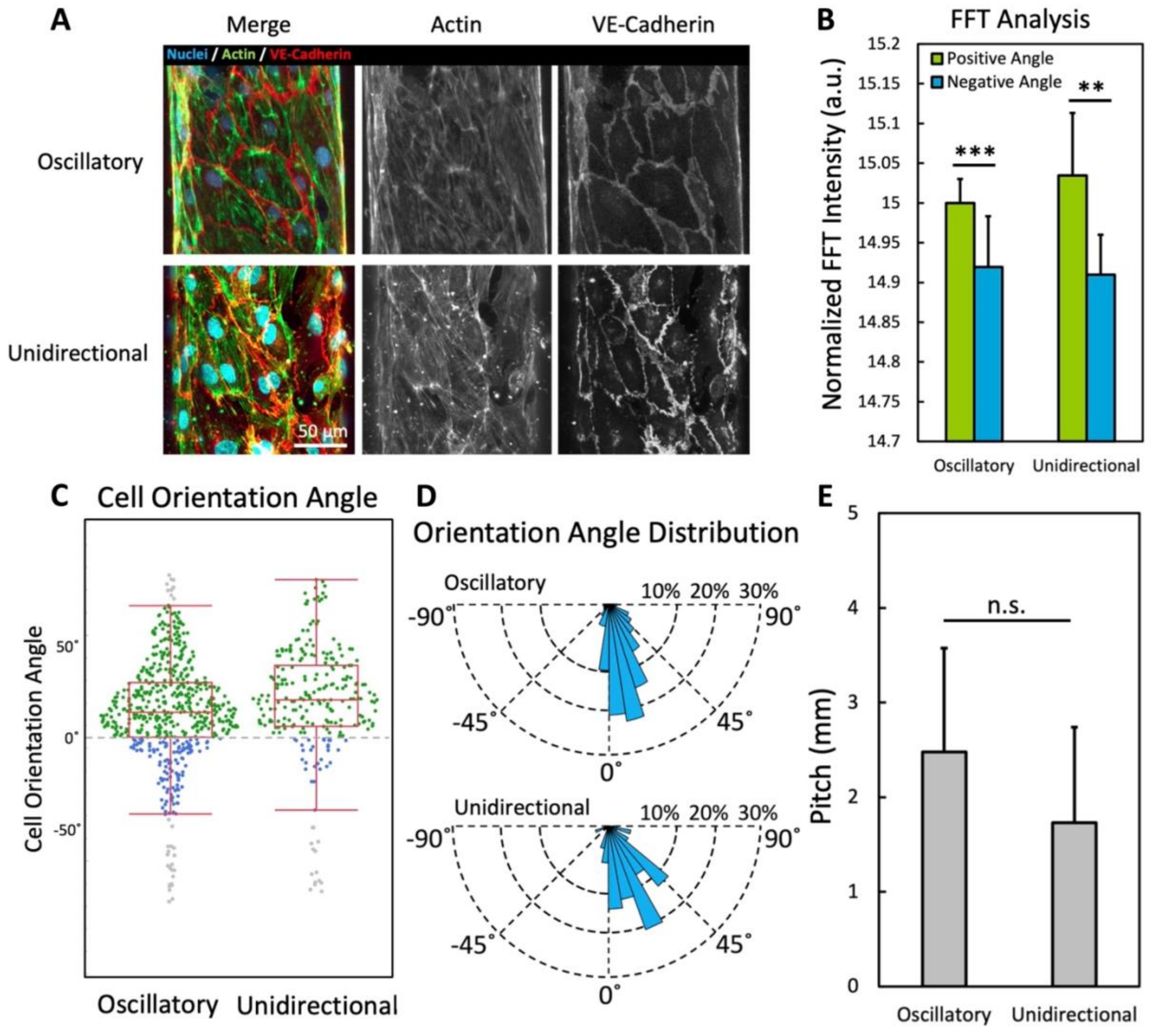

Figure S2. Effects of different flow profiles on the helical asymmetry in the *in vitro* vessels. (A) 2D projections of z-stack images of the *in vitro* vessels cultured under oscillatory or unidirectional flow. The vessels were co-stained for actin (green), nuclei (blue), and VE-Cadherin (red). The images are presented as merged, actin channel, and VE-Cadherin channel. (B) Summation of FFT intensity distributed at positive angles (5°- 80°) *vs.* negative angles (-80°- -5°) for vessels cultured under oscillatory or unidirectional flow, $p^{**}<0.01$ and $p^{***}<0.001$ by paired student's t-test, n>3 vessels per group, >5 image sets per vessel from different positions. (C) Angular distribution of cell long axis orientations for vessels cultured under oscillatory or unidirectional flow at positive angles (green dot), negative angles (blue dot), and outliers (grey dot), n>3 vessels per group, >400 cells per group. (D) Rose plot showing the radial distribution of cell long axis orientations of vessels cultured under oscillatory or unidirectional flow. (E) The

calculated helical pitch of cell alignment for vessels cultured under oscillatory or unidirectional flow (data presented as average by image sets, n>3 vessels per group, >5 image sets per vessel from different positions). n.s. represents no statistical difference by student's t-test.

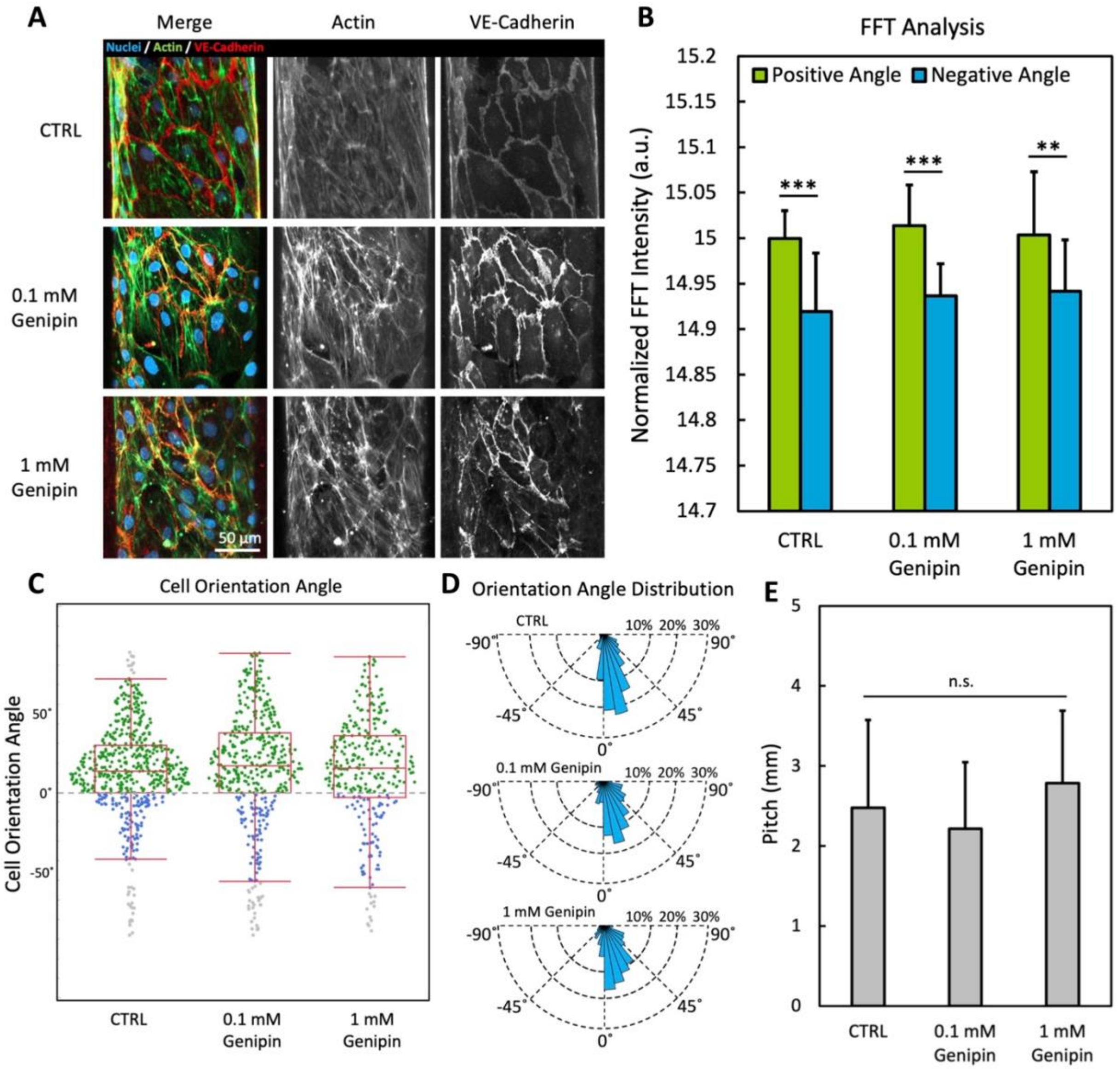

Figure S3. Effects of substrate stiffness on the helical asymmetry in the *in vitro* vessels. (A) 2D projections of z-stack images of the *in vitro* vessels with different concentrations of Genipin. The vessels were co-stained for actin (green), nuclei (blue), and VE-Cadherin (red). The images are presented as merged, actin channel, and VE-Cadherin channel. (B) Summation of FFT intensity distributed at positive angles (5° - 80°) *vs.* negative angles (-80° - -5°) for vessels with different concentrations of Genipin, $p^{**}<0.01$ and $p^{***}<0.001$ by paired student's t-test, n=5 vessels per group, >5 image sets per vessel from different positions. (C) Angular distribution of cell long axis orientations for vessels with different concentrations of Genipin at positive angles (green dot), negative angles (blue dot), and outliers (grey dot), n=5 vessels per group, >400 cells per group. (D)

Rose plot showing the radial distribution of cell long axis orientations of vessels with different concentrations of Genipin. (E) The calculated helical pitch of cell alignment for vessels with different concentrations of Genipin (data presented as average by image sets, n=5 vessels per group, >5 image sets per vessel from different positions). n.s. represents no statistical difference by one-way ANOVA with the Tukey HSD.

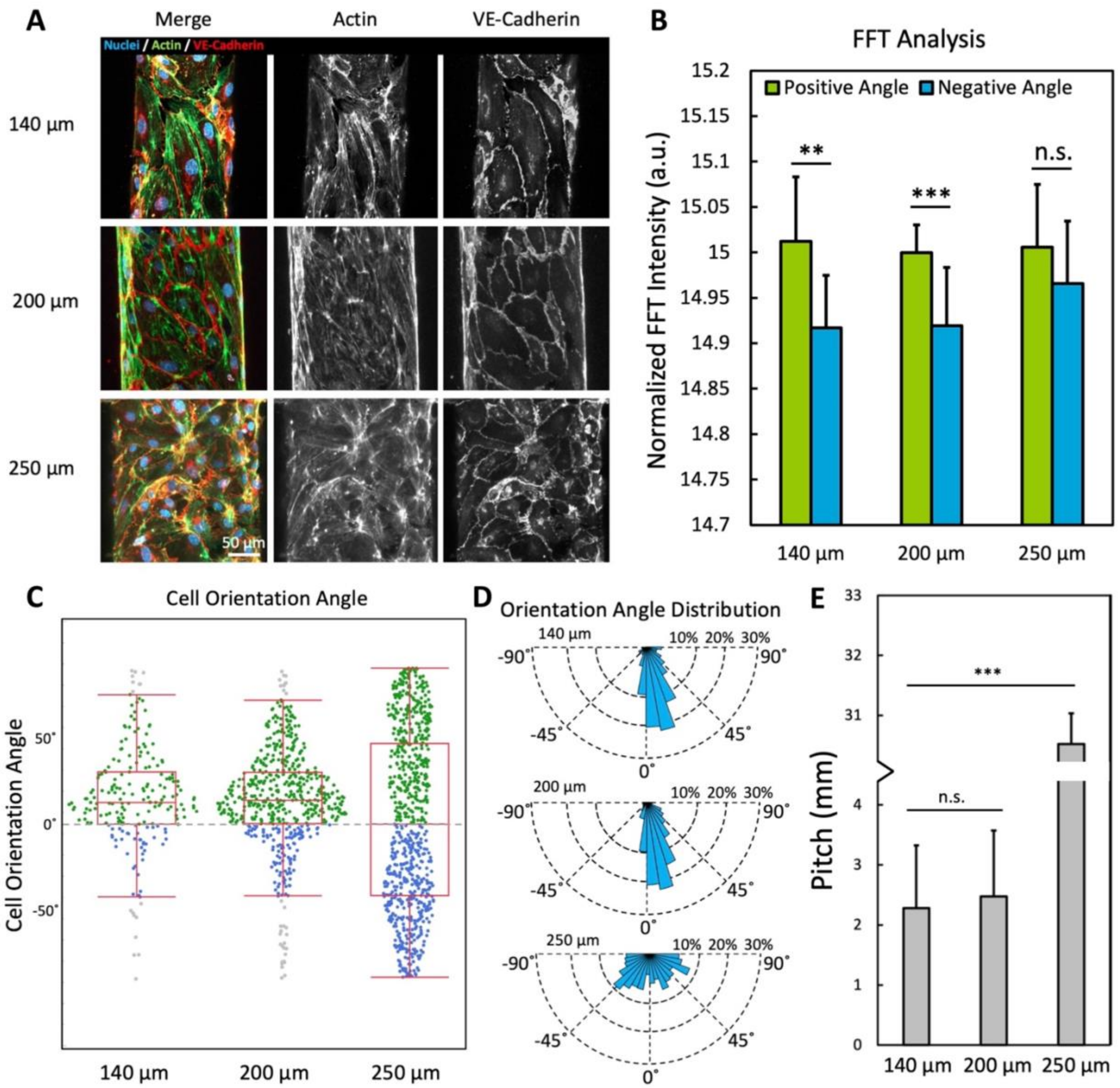

Figure S4. Effects of vascular curvature on the helical asymmetry in the *in vitro* vessels. (A) 2D projections of z-stack images of the *in vitro* vessels with different diameters. The vessels were co-stained for actin (green), nuclei (blue), and VE-Cadherin (red). The images are presented as merged, actin channel, and VE-Cadherin channel. (B) Summation of FFT intensity distributed at positive angles (5°- 80°) *vs.* negative angles (-80°- -5°) for vessels with different diameters, $p^{**}<0.01$ and $p^{***}<0.001$, n.s. represents no statistical difference by paired student's t-test, n⩾4 vessels per group, >5 image sets per vessel from different positions. (C) Angular distribution of cell long axis orientations for vessels with different diameters at positive angles (green dot), negative angles (blue dot), and outliers (grey dot), n⩾4 vessels per group, >250 cells per group.

(D) Rose plot showing the radial distribution of cell long axis orientations of vessels with diameters. (E) The calculated helical pitch of cell alignment for vessels with different diameters. (data presented as average by image sets, $n \geqslant 4$ vessels per group, >5 image sets per vessel from different positions). p***<0.001, n.s. represents no statistical difference by one-way ANOVA with the Tukey HSD.

Supplementary Video 1. 3D structure of the *in vitro* vessel shown in Figure 1. The vessel is displayed in maximum intensity projection mode.

Supplementary Video 2. 3D structure of the *in vitro* vessel shown in Figure 1. The vessel is displayed in blend mode.

Supplementary Video 3. 3D structure of the *in vitro* vessel shown in Figure 1. The vessel is displayed in normal shading mode.

Supplementary Video 4. Flat projection showing the twisting morphogenesis of the cylindrical microvessel tissue in the cell vertex model, as shown in Figure 6. The purple, thicker edges represent the top half of the tissue, and the grey, fainter-yellow edges represent the bottom half.
